# Analysis of clinical characteristics of severe pertussis in infants and children: a retrospective study

**DOI:** 10.1186/s12887-021-02507-4

**Published:** 2021-02-05

**Authors:** Caiying Wang, Huimin Zhang, Yanlan Zhang, Lin Xu, Min Miao, Hongling Yang, Yuhuan Liu, Shuxin He, Lin Pang

**Affiliations:** Department of Pediatrics, Beijing Ditan Hospital, Capital Medical University, Beijing, China

**Keywords:** pertussis, clinical characteristics, severe pneumonia, hyperleukocytosis

## Abstract

**Background:**

The incidence of pertussis shows an increasing trend in recent years, but some clinicians often lack sufficient understanding of the clinical characteristics and risk factors for severe pertussis, and more effective measures should be taken to reduce the incidence and mortality of pertussis in young infants

**Methods:**

A retrospective study was conducted, and 184 infants and children with pertussis who had been hospitalized in the Department of Pediatrics of Beijing Ditan Hospital affiliated with Capital Medical University from January 2016 to December 2017 were included. Clinical data of the patients were collected and the clinical characteristics were statistically analyzed

**Results:**

Among the 184 patients, 41.85% were infants < 3 months of age, and 65.22% of the total patients were not vaccinated against pertussis. There were 22 critically ill children, among whom 4 died, and compared with mild cases, they had a higher proportion of children younger than 3 months of age and infants not vaccinated against pertussis (63.64% vs. 38.89% and 100% vs. 60.49%, respectively); a higher proportion of children with severe pneumonia (100% vs. 0%); higher leukocyte count(× 109/L , 35.80 ± 20.53 vs 19.41 ± 8.59); and a higher proportion of children with severe hyperleukocytosis (18.18% vs. 0%, respectively) (*P*<0.05)

**Conclusions:**

1. Infants aged <3 months not vaccinated for pertussis appear more likely to become infected and have more severe disease. 2. Severe pneumonia and hyperleukocytosis are the main mechanisms underlying severe pertussis.

## Background

With the development of modern medicine, the incidence of many infectious diseases has significantly decreased, along with their threat to human health. However, the incidence of pertussis (whooping cough) shows an increasing trend. This phenomenon is known as “pertussis resurgence”. Severe pertussis can cause sudden infant death, which is not completely avoidable [1, 2]. Currently, the reported mortality rate of pertussis is 1.2–3.0% [3]. There are two circumstances contributing to this. First, this mortality occurs in part because the pathogenesis and lethality mechanisms of pertussis are not completely clear. Second, some clinicians often lack sufficient understanding of the clinical characteristics and risk factors for severe pertussis and thus do not focus their attention on these factors.

In the last few years, the number of pediatric patients with pertussis treated by our hospital has increased. This has included many severe cases and deaths. In this study, we employed a retrospective study design to analyze the clinical data of pediatric patients with pertussis treated by the department of pediatrics at Ditan Hospital in the 2 years of 2016 and 2017 in order to examine the clinical characteristics of pertussis and provide a clinical basis for the prevention and treatment of severe pertussis.

## Methods

### Study subjects

This was a retrospective study which had been approved by the ethics committee of Beijing Ditan Hospital, Capital Medical University (approval number2018-6-12B). 184 pediatric patients with pertussis, aged 0-6 years, who were treated at the Department of Pediatrics at Ditan Hospital from January 2016 to December 2017, were included as study subjects. All these children met the diagnostic criteria for pertussis (see below).

### Methods

#### The diagnostic criteria for pertussis were as follows [4]

1) 0–3 months of age: no fever or low fever, cough with increasing frequency and severity, plus one of the following: cough with the characteristic whoop, apnea, vomiting after cough, cyanosis, convulsions, pneumonia, and close contact with patient with long-term fever-free cough (usually a family member); or only paroxysmal apnea, cyanosis, and convulsions without a cough. 2) 4 months to 9 years old: no fever or low fever, paroxysmal cough ≥ 7 days, non-purulent rhinitis, and one of the following: cough with the characteristic whoop, vomiting after coughing, apnea, convulsions, pneumonia, exacerbated symptoms at night, and close contact with patient with a long-term fever-free cough. The patient should also meet at least one of the following laboratory diagnostic criteria: *Bordetella pertussis* was cultured in the laboratory, *Bordetella pertussis* was detected by polymerase chain reaction (PCR), or paired serological test was positive (immunoglobulin G (IgG) titer of single enzyme linked immunosorbent assay (ELISA) test was significantly increased > 80–100 U/ml). In this study, the pathogenic diagnosis was performed using a *Bordetella pertussis* nucleic acid detection kit, and deep sputum samples of the infants were collected for testing.

Infants with pertussis who also had hypoxemia (oxygen saturation<92%), recurrent apnea, cardiovascular dysfunction, or pertussis encephalopathy were considered to have severe pertussis. The diagnostic criteria for cardiovascular dysfunction was as follows: increased heart rate (<1year: >160/min, 1-3years: >140/min, 3-6years: >120/min); increased breathing rate(<1year: >60/min, 1-3years:>50/min, 3-6year:>40/min); cardiomegaly (by type-B ultrasonic or X ray checks); irritable, feeding difficulties, oliguresis, edema, idrosis, and breath with difficulty (satisfying at least two items). Children satisfying the above four conditions accompanied with at least one of the following conditions, or the above two conditions accompanied with at least two of the following conditions: hepatomegaly, pulmonary edema, and gallop rhythm. The diagnostic criteria for pertussis encephalopathy: infants with pertussis had hyperspasmia, and examination of cerebrospinal fluid, head MRI, and electrolytic examination of blood serum excluded central nervous system infection and other diseases that could lead to hyperspasmia.

#### Treatment

Pertussis was treated with intravenous azithromycin at a dose of 10 mg/kg body weight, once a day for 3 days. After a 4-day interval, a second treatment course was administered if spasmodic cough was still severe with the same dosage and treatment course. Azithromycin was administrated orally to neonates less than 28 days old, with the same dosage and course of treatment as above. If the infants were found to have other bacterial infections, other antibiotics were added as needed. If the infants had respiratory failure, myocardial failure, pertussis encephalopathy, or other organ damage, appropriate symptomatic supportive treatments were administrated.

### Clinical data collection and specimen collection

The patients’ age, sex, and vaccination status were noted, and blood and sputum samples were collected under sterile condition. When the sputum was too viscous for the infants to cough out, aerosol inhalation therapy with sterile saline, patting back and suctioning sputum from the trachea was performed to promote sputum discharge and to cough out, and to keep respiratory tract unobstructed. Sputum samples were collected when the above therapeutic measures were performed. The infant was fixed by parents in dorsal position with its head back, and its nasal fossae was washed twice with sterile saline. The back end of suction sputum tube was connected with vaccum extractor, and the front end was inserted into the nasal cavity of the patient, and when reaching the trachea, 0.5-1ml of sputum was extracted. The sputum sample was collected in sterile specimen-box before it was sent to the laboratory within 30 min, and the sample was used for Bordetella pertussis testing and bacterial culture, and the results might guide antibiotic treatment. Routine blood test (the test detected the count and percentage of each type of blood cell), alanine aminotransferase (ALT) and creatine kinase isozyme (CK-MB) were tested. When clinically indicated, arterial blood gas analysis was performed. Chest computed tomography (CT) and X-rays were used to evaluate the pulmonary infection status of the infants, and the complications (respiratory failure, heart failure, apnea, pertussis encephalopathy, pneumonia, liver damage, and myocardial damage) were evaluated using clinical manifestations and laboratory tests. Antibiotic use, length of hospital stay, and clinical data of routine blood test, C-reactive protein, procalcitonin, and sputum bacterial culture results were collected.

### Statistical Methods

SPSS 22.0 software was used for data analysis. Quantitative data such as age, leukocyte count, platelet count, and length of hospitalization were expressed as the mean ± SD . For comparison between moderate and severe cases, the t-test was used for normally distributed data, while the rank sum test was used for non-normally distributed data. Qualitative data such as pulmonary consolidation, fever, non-vaccination with pertussis vaccine, and C-reactive protein elevation were expressed as rates. The chi-square test was used for comparison between the two groups.

## Results

### General information

From January 2016 to December 2017, we enrolled 184 pertussis patients at our hospital, of which 102 were males and 82 were females. The youngest was 23 days old and the oldest was 4 years old. The mean age was 4.50 ± 3.95 months. 77 (41.85%) were < 3 months of age; 120 (65.22%) were not vaccinated against pertussis. 23(12.50%) were premature babies at birth, 21(11.41%) were with a low birth weight(<2500g), and 10(5.43%) were macrosomia (birth weight>4000g). 5(2.72%) had congenital heart disease, and no other co-morbidities were found. 162(88.04%) were *mild* cases, and 22 (11.96%) were severe cases who were complicated with at least one of the following: hypoxemia(18, 9.78%), heart failure(16, 8.70%), recurrent apnea(11,5.98%), or pertussis encephalopathy(3,1.63%)(Tables 1 and 2).

**Table 1:** General information of 184 pertussis patients

	Total cases *n*=184
Gender	
Male	102(55.43%)
Female	82(44.57%)
Age (months)	4.50 ± 3.95
≤1months	10(5.43%)
1-3months	67(36.41%)
3-6months	56(30.43%)
6-12months	36(19.57%)
1-4years	15(8.15%)
Not vaccinated with pertussis vaccine	120(65.22%)
Prematurity	23(12.50%)
Low birth weight	21(11.41%)
Macrosomia	10(5.43%)
Congenital heart disease	5(2.72%)
Severity	
Mild cases	162(88.04%)
Severe cases	22(11.96%)

**Table 2:** Clinical characteristics of child pertussis

	Total cases(*n*=184)
Hypoxemia	18(9.78%)
Heart failure	16(8.70%)
Recurrent apnea	11(5.98%)
Pertussis encephalopathy	3(1.63%)
Pneumonia^b^	154(83.70%)
Severe pneumonia^b^	22(11.96%)
Leukocyte count (× 10^9^/L)	23.01±14.01
Fever	53(28.80%)
Elevated neutrophilia percentage^a^	25(13.59%)
Elevated C-reactive protein^a^	31(16.85%)
Elevated procalcitonin^a^	11(5.98%)
Positive bacterial sputum culture	23(12.5%)
Antibiotic usage	
Three or more antibiotics	26(14.13%)
Special-grade antibiotics^c^	19(10.33%)
Oxygen inhalation with nasal catheter or head cover	8(4.35%)
Ventilator-assisted breathing	14(7.61%)
Length of hospitalization (days)	15.68 ± 8.11
Cases of death	4(2.17%)

^a^ The normal range of neutrophilia percentage between the age of 6 days to 4 years is 30-35%, that of C-reactive protein is <5mg/L, and that of procalcitonin is<0.25ng/ml.

^b^ Pneumonia was diagnosed when complicated with one of the following conditions: fever; cough; increased breathing rate; moist rales in the lungs, and at the same time satisfying the following condition: chest computed tomography (CT) or X-rays shows infective lesions in the lungs. Severe pneumonia was diagnosed when children with pneumonia complicated with one of the following conditions: conscious disturbance; tachypnea; apnea; oxygen saturation<92%; dehydration; chest computed tomography (CT) and X-rays shows area of lesions >2/3 of one lung, lesions in multiple lobes of two lungs, pleural effusion, pneumothorax, atelectasis, pulmonary necrosis, or pulmonary abscess; or extrapulmonary complications [5].

^c^ Special-grade antibiotics include glycopeptide and carbapenem antibiotics in this study.

### Clinical characteristics of child pertussis

Of the 184 hospitalized infants with pertussis, in addition to the serious complications named above in 22 severe cases, 83.70% of the patients had pneumonia, 11.96% had severe pneumonia. Most patients had increased white blood cell count (23.01±14.01× 10^9^/L), and the increase in white blood cells was mainly made up of lymphocytes. Over the course of the disease, 28.8% of the patients had fever, and 13.59% of them showed increased neutrophil percentage. C-reactive protein and procalcitonin increased in 16.85% and 5.98% of the patients, respectively, suggesting the presence of other bacterial infections. In addition, 12.5% had positive sputum bacterial culture. Patients using 3 or more antibiotics and special-grade antibiotics (carbapenems and glycopeptides) accounted for 14.13% and 10.33% respectively, 14 cases (7.61%) required ventilator-assisted breathing, and 8(4.35%) required oxygen inhalation with nasal catheter or head cover . 4 patients died (2.17%), which would be described in detail below. The average hospital stay was 15.68 ± 8.11 days (Table 2).

### Clinical characteristics of severe pertussis

There were 22 severe cases, and 18 of them had hypoxemia, 16 had heart failure, 11 had recurrent apnea, and 3 had pertussis encephalopathy (table 2). Compared to the children with mild pertussis, the 22 severe cases were younger, 63.64% were < 3 months of age, and the proportion of infants < 3 months of age was significantly higher than that of patients with mild pertussis. None of the severe cases had been vaccinated against pertussis, showing a significantly higher rate of non-vaccination than in the children with mild pertussis. The incidence of severe pneumonia and fever, and the percentage of children with elevated neutrophil percentage, elevated C-reactive protein and procalcitonin levels were higher, as well as the values of C-reactive protein and procalcitonin, and the positive rate of sputum bacterial culture was significantly higher than the mild cases. Moreover, the white blood cell count of severe cases was higher than those of mild cases, and the proportions of patients with severe hyperleukocytosis (white blood cells > 50*10E9/L) were also significantly higher in the severe cases than in mild cases. The length of hospitalization was significantly longer, and the proportions of using 3 or more antibiotics and special-grade antibiotics were also higher in the severe cases than in mild cases (*P*<0.05, Table 3).

**Table 3:** Comparison of clinical characteristics of the severe and mild cases

	Severe cases (*n* = 22)	Mild cases (*n* = 162)
Gender		
Male	12	90
Female	10	72
Age (months)*	2.80 ± 1.26	4.97 ± 4.30
< 3 months *	14 (63.64%)	63 (38.89%)
Not vaccinated with pertussis vaccine*	22 (100%)	98 (60.49%)
Prematurity	4(18.18%)	19(11.73%)
Low birth weight	1(4.55%)	20(12.35%)
Macrosomia	2(9.09%)	8(4.94%)
Congenital heart disease	0	5(3.09%)
Fever*	19 (86.36%)	34 (20.99%)
Severe pneumonia	22 (100%)	0
Elevated neutrophilia percentage *	15 (68.18%)	10 (6.17%)
Elevated C-reactive protein*	11 (50.00%)	20 (12.33%)
CRP value (mg/L)*	22.37±48.89	1.52±2.13
Elevated procalcitonin *	9 (40.91%))	2 (1.23%)
PCT value (ng/ml)*	1.88±4.69	0.08±0.10
Positive bacterial sputum culture*	8 (36.36%)	15 (9.26%)
Leukocyte count (× 10^9^/L)*	35.80 ± 20.53	19.41 ± 8.59
Leukocyte count > 50× 10^9^/L*	4 (18.18%)	0
Antibiotic usage		
Three or more antibiotics*	18 (81.82%)	8 (4.94%)
Special-grade antibiotics*	15 (68.18%)	4 (2.47%)
Length of hospitalization (days)*	27.71 ± 11.19	14.56 ± 5.84
Cases of death	4(18.18%)	0

*p<0.05, comparison between moderate and severe cases using statistical methods.

### Etiological analysis of pertussis-complicated pneumonia

Of the 184 children with pertussis, 23 had positive sputum bacterial culture (12.50%), 15 in the mild group and 8 in the severe group. The pathogens found in the mild group were common community infectious pathogens, such as *Staphylococcus aureus*, *Escherichia coli*, *Haemophilus influenzae*, and *Streptococcus pneumoniae*, while Gram-negative bacillary infections were more common in the severe group. These bacilli included *Acinetobacter baumannii*, *Pseudomonas aeruginosa*, *Enterobacter cloacae*, and *Klebsiella pneumoniae*. Among them, the sputum cultures of 4 severe cases were found to contain multi-drug resistant bacteria (2 cases of *Acinetobacter baumannii*, 1 case of *Pseudomonas aeruginosa*, and 1 case of *Stenotrophomonas maltophilia*), and 2 and more types of bacteria were cultured in the specimens of 3 severe cases.

### Clinical characteristics of 4 deaths

Of the 22 children with severe pertussis, 4 died, all of them were infants younger than 6 months of age and 2 younger than 3 months of age. None of them had been vaccinated with pertussis vaccine. The white blood cell count of 3 cases was > 50 × 10^9^/L, with the highest count of 106.68 × 10^9^/L. All 4 cases were complicated with severe pneumonia, the percentage of neutrophils was high (44.00–81.54%), C-reactive protein was higher than normal in 3 cases (21.1–235.5 mg/L, with normal value of < 5 mg/L), and procalcitonin was high in 2 cases (1.05–8.22 ng/ml, with normal value of < 0.25 ng/ml). All had fever during the course of the disease. One case had positive sputum bacterial culture, which was *Enterobacter aerogenes*. The drug sensitivity test of the *Bordetella pertussis* isolated from a 56-day-old infant showed resistance to macrolides. The causes of death of 2 were respiratory and heart failure, 1 was heart failure, and 1 was acute respiratory distress syndrome (ARDS). (Table 4)

**Table 4:** Clinical characteristics of 4 deaths

	No.1	No.2	No.3^a^	No.4
Age (months)	2.90	5.20	1.87	4.00
Gender	female	male	female	male
Pertussis vaccination	no	no	no	no
Severe pneumonia	yes	yes	yes	yes
Fever	yes	yes	yes	yes
Leukocyte count (× 10^9^/L)	58.2	29.8	50.98	106.68
Neutrophilia percentage	44.66%	70.04%	81.54%	44.00%
C-reactive protein (mg/L)	0	23.90	235.50	21.10
Procalcitonin (ng/ml)	0.05	8.22	1.05	0.09
Bacterial sputum culture	negative	negative	negative	*Enterobacter aerogenes*
Length of hospitalization (days)	5	10	21	3
Causes of death	heart failure	respiratory and heart failure	ARDS	respiratory and heart failure

This table shows the general information and clinical data of 4 deaths in severe pertussis cases.

^a^ The drug sensitivity test of the *Bordetella pertussis* isolated from this patient showed resistance to macrolides

## Discussion

Pertussis can occur in all age groups, with the highest incidence in infants and young children, and infants under 1 year of age have relatively more severe disease. The infants included in this study were hospitalized pertussis patients, with an average age of 4.50 ± 3.95 months. Most of them were young infants with relatively severe conditions. In this study, 65.22% of the infants were not vaccinated against pertussis, and 41.85% were infants younger than 3 months. None of the severe infants were vaccinated, of whom 63.64% were younger than 3 months of age. The study shows that nearly half of the patients experienced onset of pertussis before the standard age of pertussis vaccination in China (3 months of age), and the proportion of unvaccinated infants was significantly higher in the severe cases than in the mild cases, which was consistent with the results of previous studies [6]. In order to decrease the rate of pertussis overall and reduce the number of severe cases, the government should consider earlier pertussis vaccination, as WHO recommended infants received pertussis vaccination at 6 weeks, or to vaccinate pregnant women to produce protective effects for the infants. Because IgG antibodies can cross the placenta, almost all IgG antibodies present in infants 6 months old and younger arise from the mother. A number of studies showed that when acellular pertussis vaccine was given to pregnant women in the second or third trimester, especially at 27–36 weeks of pregnancy, enough antibodies against Bacillus pertussis, such as the anti-pertussis toxin and anti-filament hemagglutinin antibodies, were detected in the blood of the neonates, and the antibodies persisted and gradually declined until completely attenuated at the age of 3 months [7–9]. Therefore, acellular pertussis vaccine for pregnant women could protect infants from pertussis infections before the standard age of pertussis vaccination (the age of 3 months). So far, acellular pertussis vaccination in pregnancy has been implemented in Brazil, Argentina, UK, USA, Belgium and New Zealand. An observational study showed that this strategy conferred up to 90% protection for infants under 3 months from pertussis, by the dual effect of preventing maternal infection thereby minimizing mother-to-baby transmission, and also by virtue of specific IgG antibodies from the mother crossing the placenta affording protection to baby after birth [10–13]. In Argentina the deaths of pertussis cases deceased by 87% in 2013 compared to 2011, since the country implemented the strategy of acellular pertussis vaccination in pregnancy in 2012 [14].

Pneumonia is the most common complication of pertussis. In this study, more than 80% of hospitalized infants with pertussis also had pneumonia. Some infants had fever, with increased neutrophils, C-reactive protein, and procalcitonin levels, suggesting the presence of other bacterial infections. All severe cases had severe pneumonia, and all of them required some form of respiratory support. In this way, pneumonia is not only the most common complication of pertussis but also the leading cause of severe disease and death from pertussis [15–17].

Increased white blood cell count is a characteristic manifestation of pertussis. The white blood cell count of children in the severe cases was significantly higher than that of the mild cases, and the white blood cell count was particularly high in fatal cases. Due to the poor ability of white blood cells to change shape, they tend to obstruct stenosed alveolar capillary beds. White blood cells usually take 10-15 times longer than erythrocytes to pass through these vessels, resulting in embolism due to leukocyte clumps, causing hypoxemia and pulmonary hypertension [18, 19]. This affects cardiac function and causes heart failure in severe cases. Severe hyperleukocytosis (> 50 × 10^9^ leukocytes/L) is an independent risk factor for malignant pertussis (life-threatening severe pertussis) [20, 21]. Many studies have indicated that when routine treatment was given even though the peripheral leukocyte count was > 100 × 10^9^/L in pertussis patients, and no measures to lower leukocyte counts were carried out, death resulted in all cases [22, 23]. In this study, there were 4 infants the in the severe group whose white blood cell counts were > 50×10E9/L, of whom 3 died. Studies have shown that plasma exchange and leukapheresis can reduce leukocyte count and significantly improve fatal hypoxemia and pulmonary hypertension [24, 25].

Pertussis not only seriously endangers the life and health of infants, but also creates a heavy burden on families and society. Severe pneumonia and hyperleukocytosis are key issues in the prevention and treatment of severe pertussis. Adjusting the immunization strategy to vaccinate pregnant women has a clear effect on reducing the incidence and mortality of pertussis in young infants, which should be considered by the government.

As a retrospective study, the study has some kind of uncontrollability, some significative data may be absent. For example, hyperleukocytosis causes pulmonary hypertension which affects cardiac function and may leads to life-threatening severe pertussis, but pulmonary arterial pressure has not been measured in these infants. In addition, a proportion of infants with pre-existing co-morbidities were included in the study, such as preterm or low birth weight infants, macrosomia, and infants with congenital heart disease, and they were at a higher risk to develop severe disease when having an infection due to the lower amount of maternal antibodies that were mainly transferred in the last trimester of pregnancy, or the immaturity or low function of their immune system. But the proportion of infants with these co-morbidities had no statistical difference between infants with severe and mild pertussis in the study, this was probably because the number of these infants was too small. The issues not covered in this study need to be studied later.

## Conclusions

1. Infants aged <3 months not vaccinated for pertussis appear more likely to become infected and have more severe disease. Adjusting the immunization strategy to vaccinate pregnant women to protect the infants should be considered.

2. Severe pneumonia and hyperleukocytosis are the main mechanisms underlying severe pertussis.

## Data Availability

The datasets used and/or analysed during the current study are available from the corresponding author on reasonable request.

## Abbreviations

PCR: Polymerase chain reaction
IgG: Immunoglobulin G
ELISA: Enzyme linked immunosorbent assay
ALT: Alanine aminotransferase
CK-MB: Creatine kinase isozyme
CT: Computed tomography
ARDS: Acute respiratory distress syndrome
